# Deep sequencing of HBV pre-S region reveals high heterogeneity of HBV genotypes and associations of word pattern frequencies with HCC

**DOI:** 10.1371/journal.pgen.1007206

**Published:** 2018-02-23

**Authors:** Xin Bai, Jian-an Jia, Meng Fang, Shipeng Chen, Xiaotao Liang, Shanfeng Zhu, Shuqin Zhang, Jianfeng Feng, Fengzhu Sun, Chunfang Gao

**Affiliations:** 1 Centre for Computational Systems Biology, School of Mathematical Sciences, Fudan University, Shanghai, China; 2 Institute of Science and Technology for Brain-Inspired Intelligence, Fudan University, Shanghai, China; 3 Molecular and Computational Biology Program, Department of Biological Sciences, University of Southern California, Los Angeles, California, United States of America; 4 Department of Laboratory Medicine, Eastern Hepatobiliary Surgery Hospital, Second Military Medical University, Shanghai, China; 5 Department of Laboratory Medicine, the 105th Hospital of PLA, Hefei, China; 6 School of Computer Science and Shanghai Key Lab of Intelligent Information Processing, Fudan University, Shanghai, China; 7 Shanghai Key Laboratory for Comtemporary Applied Mathematics, Fudan University, Shanghai, China; 8 Department of Computer Science, University of Warwick, Coventry, United Kingodm; National Institute of Genetics, JAPAN

## Abstract

Hepatitis B virus (HBV) infection is a common problem in the world, especially in China. More than 60–80% of hepatocellular carcinoma (HCC) cases can be attributed to HBV infection in high HBV prevalent regions. Although traditional Sanger sequencing has been extensively used to investigate HBV sequences, NGS is becoming more commonly used. Further, it is unknown whether word pattern frequencies of HBV reads by Next Generation Sequencing (NGS) can be used to investigate HBV genotypes and predict HCC status. In this study, we used NGS to sequence the pre-S region of the HBV sequence of 94 HCC patients and 45 chronic HBV (CHB) infected individuals. Word pattern frequencies among the sequence data of all individuals were calculated and compared using the Manhattan distance. The individuals were grouped using principal coordinate analysis (PCoA) and hierarchical clustering. Word pattern frequencies were also used to build prediction models for HCC status using both K-nearest neighbors (KNN) and support vector machine (SVM). We showed the extremely high power of analyzing HBV sequences using word patterns. Our key findings include that the first principal coordinate of the PCoA analysis was highly associated with the fraction of genotype B (or C) sequences and the second principal coordinate was significantly associated with the probability of having HCC. Hierarchical clustering first groups the individuals according to their major genotypes followed by their HCC status. Using cross-validation, high area under the receiver operational characteristic curve (AUC) of around 0.88 for KNN and 0.92 for SVM were obtained. In the independent data set of 46 HCC patients and 31 CHB individuals, a good AUC score of 0.77 was obtained using SVM. It was further shown that 3000 reads for each individual can yield stable prediction results for SVM. Thus, another key finding is that word patterns can be used to predict HCC status with high accuracy. Therefore, our study shows clearly that word pattern frequencies of HBV sequences contain much information about the composition of different HBV genotypes and the HCC status of an individual.

## Author summary

HBV infection can lead to many liver complications including hepatocellular carcinoma (HCC), one of the most common liver cancers in China. High-throughput sequencing technologies have recently been used to study the genotype sequence compositions of HBV infected individuals and to distinguish chronic HBV (CHB) infection from HCC. We used NGS to sequence the pre-S region of a large number of CHB and HCC individuals and designed novel word pattern based approaches to analyze the data. We have several surprising key findings. First, most HBV infected individuals contained mixtures of genotypes B and C sequences. Second, multi-dimensional scaling (MDS) analysis of the data showed that the first principal coordinate was closely associated with the fraction of genotype B (or C) sequences and the second principal coordinate was highly associated with the probability of HCC. Third, we also designed K-nearest neighbors (KNN) and support vector machine (SVM) based classifiers for CHB and HCC with high prediction accuracy. The results were validated in an independent data set.

## Introduction

The hepatitis B virus (HBV) is a DNA virus infecting around 257 million people worldwide (http://www.who.int/mediacentre/factsheets/fs204/en/) and can cause liver diseases and hepatocellular carcinoma (HCC), one of the most common types of liver cancer [1, 2]. About 500,000 HBV patients die each year worldwide from HBV related complications and about 10% of the HBV infected individuals will have HCC during their life time [3]. However, the understanding of the differences of HBV compositions based on next generation sequencing (NGS) technologies between chronic hepatitis B (CHB) and HBV related HCC is limited.

The HBV sequences are currently divided into 10 HBV genotypes, A to J, with genome wide differences of 8%, and 35 subgenotypes using genome wide differences of 4% [3–5]. HBV genotypes have been shown to be associated with geographical locations [6, 7]. In China, the most common genotypes are B and C [8, 9]. Besides, some individuals can be infected by viruses of multiple genotypes and there can be some recombinations among the different genotypes. Different genotypes have varied effects on disease severity, course and likelihood of complications, response to treatment and possibly vaccination [10, 11]. It has been shown that genotype C is associated with more disease complications and higher chance of HCC transition than genotype B [12].

Due to the high mutation rate of the HBV and the possibility of multiple HBV infections, there are high inter- and intra- patient HBV geneticdiversities. Previous studies revealed that basal core promoter (BCP) A1762T/G1764A mutations were strongly associated with the occurrence of HCC [13–16]. Truncated large surface proteins due to deletions in the pre-S gene were observed to accumulate in the endoplasmic reticulum (ER), resulting in ER stress and hepatocarcinogenesis [17, 18]. It was also shown that some pre-S deletions or mutations were risk factors for the development of liver cirrhosis and HCC [19–22]. Meta-analysis studies indicated that pre-S deletion mutations and BCP double mutations were associated with HCC risk [13, 23–25]. Several studies have found that combination of mutations in the HBV genome could predict HCC occurrence more accurately than individual mutations [26–28].

Traditionally, only the dominant genotypes and haplotypes within the patients were investigated due to the technological limitations of Sanger sequencing that are usually time consuming and economically expensive to sequence a large number of sequences within individuals. With the development of high-throughput NGS technologies, it is now possible to investigate the HBV genetic diversity within individuals carefully and to develop more sophisticated and robust prediction models for predicting HCC.

In this study, we aim to explore the diversity of HBV pre-S sequences within HCC and CHB patients, to identify their differences, and to establish prediction models for HCC with machine learning methods based on word pattern frequencies. In detail, we first carried out a large scale HBV pre-S region study of 94 HCC patients and 45 chronic HBV infected individuals. The heterogeneity of HBV composition and the HBV genotype fraction in individuals were investigated. We used a novel alignment-free method based on word pattern frequencies to cluster the individuals and investigated the cluster distributions of HCC patients and CHB individuals. We further applied K-nearest neighbors (KNN) and support vector machine (SVM) approaches to predict HCC status based on word counts and the predictive model was validated using an independent data set consisting of 46 HCC patients and 31 CHB individuals. The key novelties of this study are the use of word patterns for the analysis of HBV sequences to cluster HBV infected individuals and to predict HCC status. Our study clearly showed the surprising high power of word patterns for clustering HBV genotypes and predicting HCC status.

## Results

### Most individuals have mixtures of genotypes B and C HBV sequences

**Fig 1:**
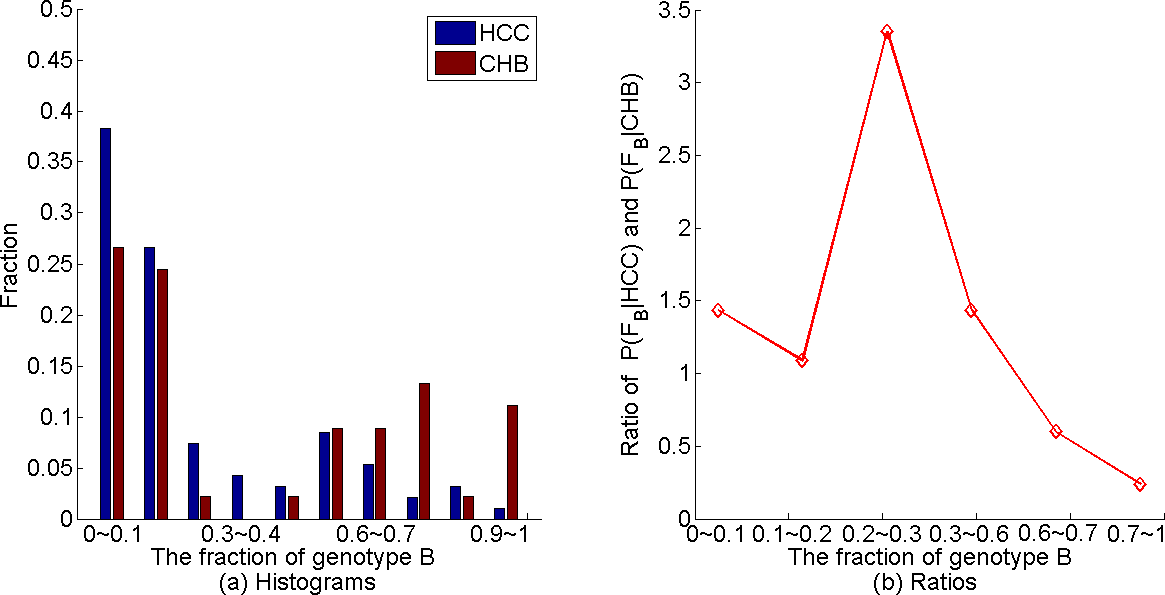
Fraction of genotype B among the 94 HCC patients and 45 CHB patients. (a) Histograms of the fraction of genotype B based on STAR. (b) The relationship between the ratio of the fraction of HCC individuals in the bin over that of the CHB individuals and the fraction of genotype B sequences based on STAR.

We genotyped each sequence in the NGS data using STAR [49] and calculated the fraction of genotypes B and C sequences for every individual as described in the “Materials and methods” section. The fraction of recombinants in 95% of the individuals (132/139) was less than 5% and most of the reads were of genotype B or C (S1 Supplementary material S1 Fig). Therefore, we ignored the recombinant reads and the reads of other genotypes and concentrated on the reads of genotype B or C in all the individuals. The histograms of the fractions of genotype B sequences among the 94 HCC patients and 45 CHB individuals are given in Fig 1(A). It can be seen from the figure that most individuals have both genotypes B and C sequences for both HCC and CHB individuals. The fraction of genotype B sequences among HCC patients has a tendency to be lower than that for the CHB individuals, consistent with previous observations that genotype C individuals are more likely to have HCC than genotype B individuals [29]. About 70% of the HCC patients have genotype B fraction less than 30% and only about 50% of the CHB patients have genotype B fraction less than 30%. While about 37% of the CHB individuals have genotype B fraction at least 70%, only about 5% of the HCC patients have genotype B fraction at least 70%.

Based on our data, we further investigated the relationship between having HCC and the fraction of genotype B in an individual. It can be shown that the probability of having HCC for given genotype B fraction increases with the ratio of fraction of individuals having the given genotype B fraction among HCC patients over that of CHB patients. Therefore, we binned both the HCC and CHB individuals according to the genotype B fraction. For each bin, we calculated the fractions HCC and CHB individuals and then calculated their ratio as shown in Fig 1(B). When the number of occurrences in a bin was small, the estimated fraction was not reliable. Thus, we required that the fractions for both HCC and CHB in each bin to be at least 5%. If either the HCC fraction or the CHB fraction in an interval was smaller than 5%, we merged it with the later intervals until both fractions were above 5%. Therefore, we merged the bins 0.3~0.4, 0.4~0.5, and 0.5~0.6 into one bin when we calculated the ratio of the fractions. Similarly, we merged the bins 0.7~0.8, 0.8~0.9 and 0.9~1.0 to form another bin. As we can see from Fig 1B that this fraction is higher than 1.0 when the fraction of genotype B sequences is less than 0.6, while it is much less than 1 when the fraction of genotype B sequences is above 0.6.

**Fig 2:**
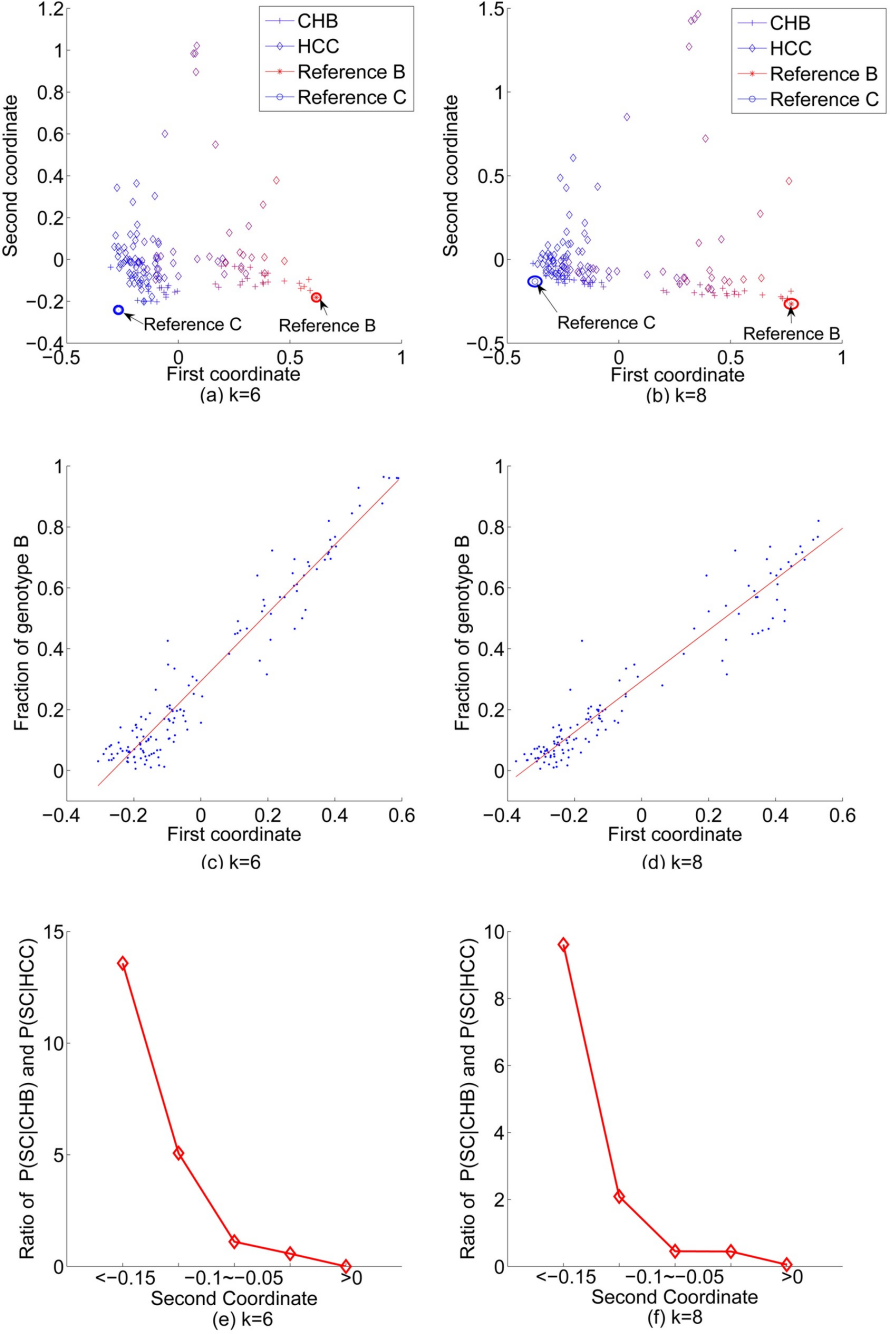
PCoA plot based on the 94 HCC patients and 45 CHB individuals. The distance matrix is based on the Manhattan distance between the frequency vectors of word patterns of length (a) k = 6 and (b) k = 8, respectively. Color shows the fractions of genotype B and C based on the STAR genotyping results. Red represents 100% genotype B and blue represents 100% genotype C. Reference B and C sequences are also added on the figures as references. The relationship between the first principal coordinate and the fraction of genotype B, (c): k = 6, (d): k = 8. The relationship between the ratio of the fraction of CHB individuals in the bin over that of the HCC individuals and the second coordinate, (e): k = 6, (f): k = 8.

**Fig 3:**
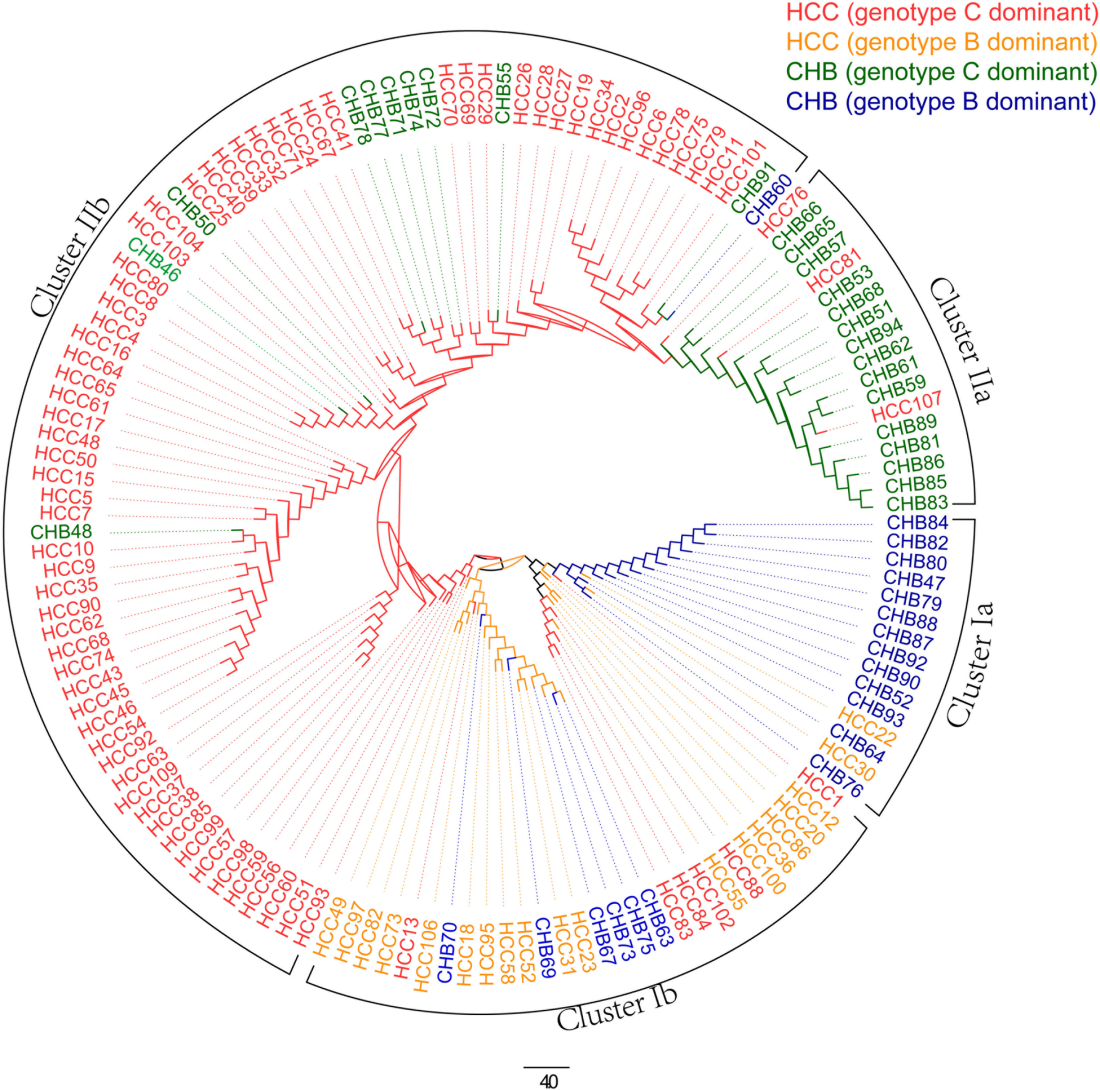
Hierarchical clustering results of samples HCC and CHB from the first data set. There are four different colors of branches: red means HCC samples genotype C dominant, yellow means HCC samples genotype B dominant, green means CHB samples genotype C dominant, blue means CHB samples genotype B dominant. One genotype dominant means the fraction of this genotype is the highest among all genotypes.

To see how genotyping method would affect the results, we also used another genotyping program, jpHMM [30], to genotype the reads. The histogram of the fraction of recombinant reads for the 139 individuals is shown in FigS2a in the S1 Supplementary material. The fraction of genotype B using jpHMM is highly associated with that based on STAR (Pearson correlation coefficient = 0.9968 and p-value = 1.0e-151) as shown in FigS2b) in the S1 Supplementary material. FigS3 in S1 Supplementary material shows a similar figure as Fig 1 when jpHMM was used for genotyping. Again we see that the probability of having HCC increases with the fraction of genotype C sequences based on jpHMM.

### Individuals mainly cluster by their HBV genotypes followed by the HCC status

Based on the word pattern frequencies of the NGS reads from the HBV pre-S region for the individuals, we used Manhattan distance to calculate the dissimilarity between any pair of individuals. We then used principal coordinate analysis (PCoA) to project the individuals onto two-dimensional Euclidean space. Fig 2A and 2B show the PCoA results for the 94 HCC patients and 45 CHB individuals using word length *k* = 6 and *k* = 8, respectively.

To see the relationship between the PCoA results and the fraction of genotype B or C in the NGS data of the HBV pre-S sequences, we colored the points corresponding to the individuals according to the fractions of B and C genotypes with red indicating 100% genotype B and blue indicating 100% genotype C with intermediate color in between based on the STAR genotyping results. We also downloaded the HBV genotypes B and C reference sequences from NCBI (accession number of genotype B: D00329, AB073846, AB602818; genotype C: X04615, AY123041, AB014381) and used the pre-S region to serve as references. We counted the occurrences of word patterns of these sequences, calculated their dissimilarity with the 139 samples, and plotted the 141 samples in the PCoA figure. We have several observations from Fig 2. First, the fraction of genotype B sequences in each individual is highly associated with the values of the first principal coordinate. From left to right of the figures, the fraction of genotype B sequences increases with the first coordinate. To see this pattern more clearly, we plotted Fig 2C and 2D that show the relationship between the first principal coordinate and the fraction of genotype B using *k* = 6 and *k* = 8, respectively. The Pearson correlation coefficient (PCC) between the fraction of genotype B sequences and the first principal coordinate is as high as 0.97 when *k* = 6 and *k* = 8. Second, the HCC tumor samples are distributed more broadly on the PCoA plots and are more diverse than the CHB individuals. The second principal coordinate seems to be associated with the HCC status with high second PCoA coordinate indicating high probability of HCC. Although the second principal coordinates for most of the CHB individuals are at similar levels as for the reference genotypes B and C sequences, many HCC samples have much higher second principal coordinate. To see the pattern more clearly, we divided the second coordinate into 5 bins: < −0.15; −0.15~−0.1; −0.1~−0.05; −0.05~0; > 0. In each bin, we calculated the fractions of CHB and HCC individuals in the bin. We also calculated their ratio and plot the relationship between the ratio and the second coordinate in Fig 2E and 2F. It can be seen that when the second coordinate is smaller than -0.1, the fraction of CHB individuals dominates and with the increase of second coordinate, the fraction of HCC individuals increases. When the second coordinate is bigger than 0, there are no CHB individuals. On the other hand, some of the HCC patients and CHB individuals mix together in the principal coordinate plots and there is no clear separation for HCC patients and CHB individuals. The above conclusions are consistent for both *k* = 6 and *k* = 8.

Fig 2 shows that the first principal coordinate is highly associated with the fractions of genotype B(C) when intuitively choosing *k* = 6 and *k* = 8. Therefore, we chose the word length *k* to maximize the correlation. Table 1 shows the Pearson and Spearman correlations between the first principal coordinate and the fraction of genotype B sequences for word length *k* ranging from *k* = 2 to *k* = 8.

**Table 1:** Spearman and Pearson correlations coefficients between the first principal coordinate and the fraction of genotype B for the 94 HCC patients and 45 CHB individuals. Different word lengths are used for computing the Manhattan distance.

Correlation	*k* = 2	*k* = 3	*k* = 4	*k* = 5	*k* = 6	*k* = 7	*k* = 8
Spearman	-0.39	0.20	0.38	0.80	0.89	0.94	0.94
Pearson	-0.37	0.19	0.42	0.92	0.97	0.97	0.97

Both the Spearman and the Pearson correlation coefficients increase with word length *k*. When *k* ≥ 6, the PCC becomes stable. Note that for *k* = 6 the correlation is already very high and considering computational efficiency, we use *k* = 6 to show our results on the training data in the rest of the paper.

In addition to the PCoA plots, we also grouped the individuals using hierarchical clustering with UPGMA (Un-weighted Pair Group Method with Arithmetic Mean) to calculate the distance between two clusters. We used the distance matrix calculated from Manhattan distance with *k* = 6 and input it into the software Mega (http://www.megasoftware.net/). Fig 3 shows the clustering results and the genotypes are analyzed using STAR. The corresponding results using jpHMM are given in S1 Supplementary material Fig S5. The individuals are generally divided into two main clusters. Cluster I contains 44 individuals, 38 of them with dominant genotype B and cluster II contains 95 individuals, 94 of them with dominant genotype C. The overlaps between the two clusters and groups of individuals with genotypes B or C are given in Table 2. The clusters are significantly associated with the dominant individual genotypes (p-value = 2.2e-16, χ2-test). Six individuals (HCC1, HCC13, HCC83, HCC84, HCC88, and HCC102) out of 101 (76HCC+25CHB) with dominant genotype C belong to cluster I. Their corresponding fractions of genotype B are 0.49, 0.49, 0.18, 0.27, 0.14, 0.29, respectively. On the other hand, only one individual (CHB60) out of 38 (18HCC+20CHB) with dominant genotype B belong to the second cluster and its fraction of genotype B is 0.59. We can see that the mis-clustered individuals are highly mixed, and their secondary genotypes also have relatively high fraction. The normalized fractions of genotypes B and C sequences of all individuals using STAR and jpHMM are given in the S1 Table.

**Table 2:** Distribution of patients according to genotype fraction and clusters. Number of overlaps between the clusters (I and II) and groups of individuals with dominant genotypes B and C, respectively.

	cluster I	cluster II
Genotype B dominant	38	1
Genotype C dominant	6	94

Within cluster I, there is a small sub-cluster Ia that is dominated by CHB individuals. On the other hand, the HCC patients and CHB individuals are not clearly separated in cluster I. Within cluster II, a small cluster IIa is dominated by CHB individuals and the HCC patients are generally far away from this group. The results from the hierarchical clustering of the individuals are consistent with the observations based on PCoA results.

We noticed 11 CHB patients within the large cluster IIb that contains mostly HCC patients. Therefore, we checked the meta-data to see if these 11 individuals had high risk factors for HCC including liver cirrhosis, advanced age, male sex, etc. Six out of the 11 CHB patients in cluster IIb had meta-data available. Five patients (CHB46, CHB48, CHB50, CHB60, CHB91) are male and one is over 60. Patient CHB55 is female, who has liver cirrhosis and was over 60 years old. Thus, our meta-data do show that these patients have more risk factors.

**Fig 4:**
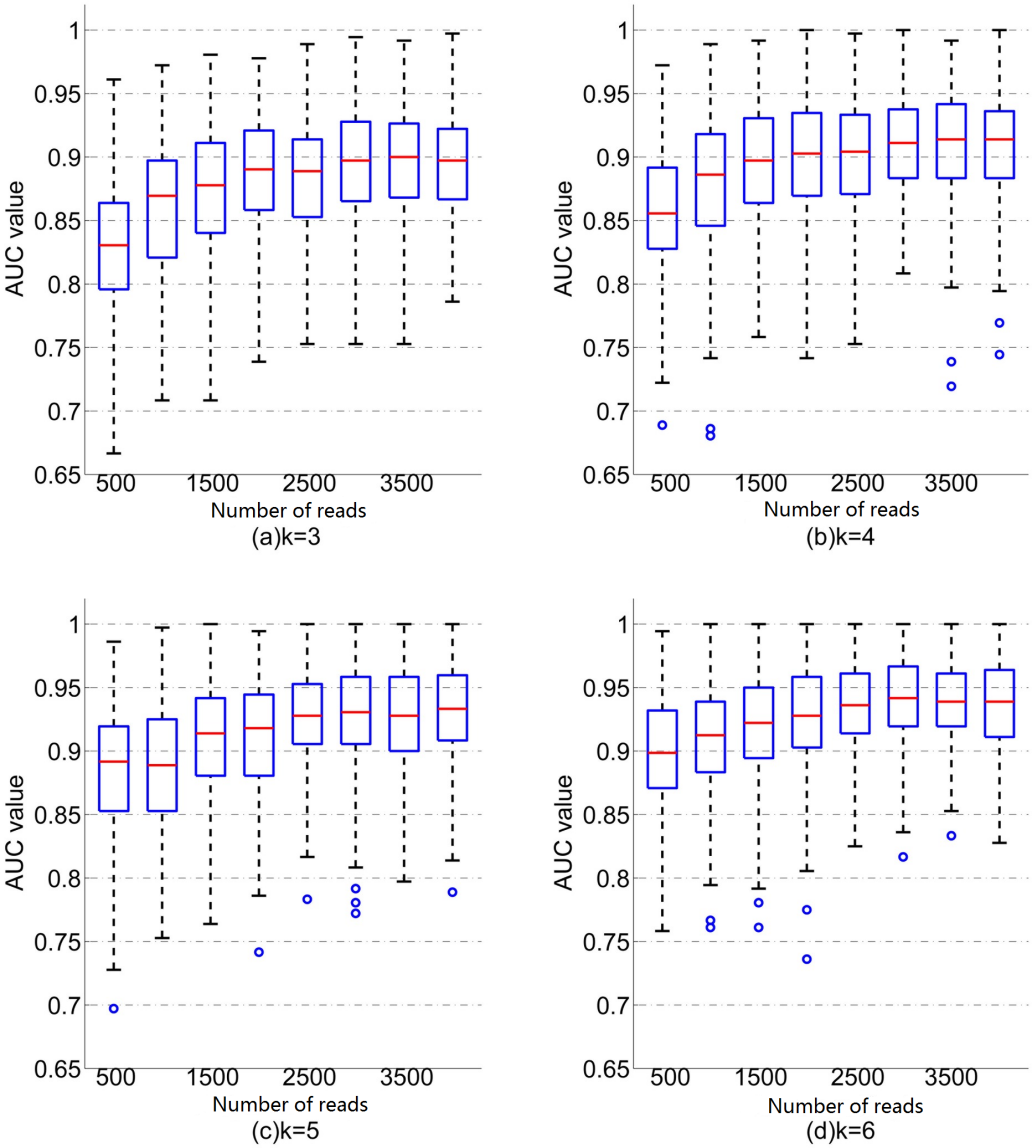
Boxplots of the relationship between AUC values and the number of readsusing different word length k for SVM. For each word length k and number of reads N, there are 200 random replicates and AUC values.

We also colored the points in the PCoA plots corresponding to the individuals according to the fractions of B and C genotypes with red indicating 100% genotype B and blue indicating 100% genotype C with intermediate color in between based on the jpHMM genotyping results, and the corresponding figure is shown as FigS4 in the S1 Supplementary material. Similar observations as based on STAR genotyping were obtained. Table S2 in the S1 Supplementary material shows again that the first principal coordinate is highly associated with the fraction of B genotypes in an individual, consistent with the results using the STAR genotyping tool.

### Prediction of HCC status within the training set and validations using an independent dataset

We used two methods, K-nearest neighbors and support vector machine (SVM), to predict HCC status based on the word pattern frequency vector of the HBV pre-S region of the samples.

The prediction results based on KNN are given in Table 3. It can be seen from the table that the cross validation results measured by AUC are roughly the same with different word length *k* and the AUCs center around 0.88. For the independent test data, the AUC increases slightly with the word length from 0.62 for *k* = 2 to 0.67 when *k* is between 6 and 8.

**Table 3:** Prediction results from KNN using different word length *k*. *CV: cross validation.

Word length *k*	*k* = 2	*k* = 3	*k* = 4	*k* = 5	*k* = 6	*k* = 7	*k* = 8
CV mean AUC	0.86	0.87	0.87	0.88	0.88	0.89	0.89
Predicting AUC	0.62	0.64	0.66	0.65	0.67	0.67	0.67
Optimal K	15	10	5	5	5	5	5

The AUC values of SVM using cross validation and testing set and corresponding parameter C using different word length *k* are shown in Table 4. We observe from the table that the prediction accuracy measured by AUC with cross-validation increases slightly with word length from 0.86 when *k* = 2 to 0.93 when *k* = 7. On the other hand, the AUC for the independent data decreases with word length from 0.77 when *k* = 3 to 0.70 when *k* = 8. When *k* = 2, the AUC is only 0.65. The good performance of the SVM model when *k* = 3 may be due to the relatively small number of learning samples such that the derived SVM model with small number of word patterns is more stable.

**Table 4:** Prediction results from SVM using different word length *k*. *CV: cross validation.

Word length *k*	*k* = 2	*k* = 3	*k* = 4	*k* = 5	*k* = 6	*k* = 7	*k* = 8
CV mean AUC	0.86	0.90	0.91	0.93	0.93	0.93	0.92
Predicting AUC	0.65	0.77	0.72	0.70	0.70	0.70	0.70
Optimal *C*	16384	16384	32768	32768	32768	32768	16384

#### Using a subset of words decreases the prediction accuracy in the independent data

We also investigated the use of a subset of all the words for predicting HCC status. For each word of length *k*, we calculated its fractions in the 45 CHB and 95 HCC training samples. We then used rank sum test statistic to test whether the two populations have the same distribution and a *p*-value was obtained. We sorted the *p* -values of all words of length *k* in ascending order. For a threshold *α*, we select the words with *p*-value less than *α*/*N*, where *N* = 4^*k*^/2 for odd *k* and *N* = (4^*k*^ + 2^*k*^)/2 for even *k* because we simultaneously considered a word and its complement for word counting. We used such a criterion based on the idea of Bonferroni correction for multiple hypothesis testing. In our study, we let *α* = 0.05, 0.01, 0.001. We only used the selected words based on training set to 1) train models and predict the HCC status for SVM, and 2) calculate the Manhattan distance for KNN. The results are presented in Tables S3-S8 in the S1 Supplementary material. Although selecting subsets of words can give better results for cross-validation prediction, the results for independent data prediction are worse. Significant words of training and testing sets could be different, which results in the importance of using all words.

#### Prediction accuracy increases with the number of reads and is stable above 3000 reads per sample

**Fig 5:**
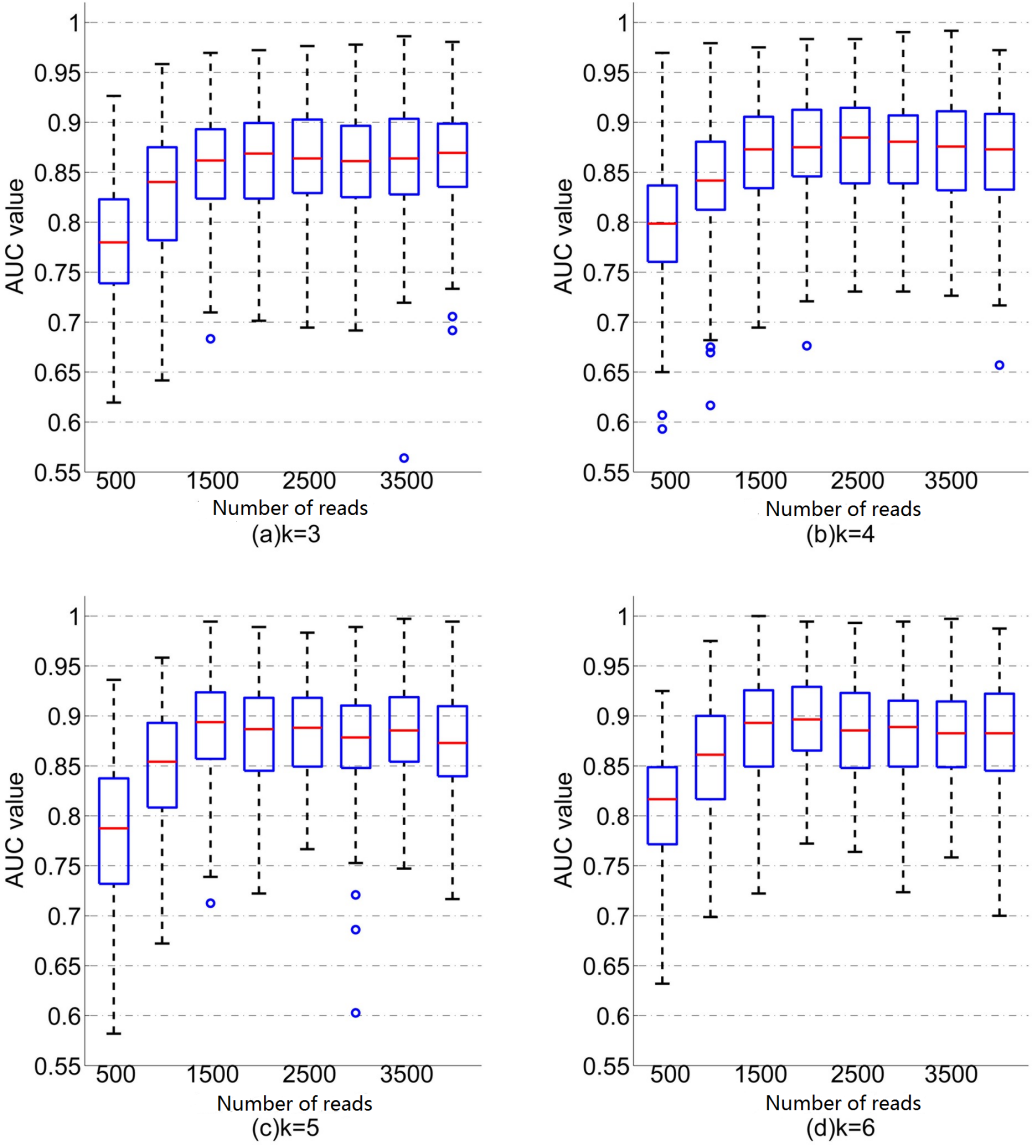
Boxplots of the relationship between AUC values and the number of readsusing different word length k for KNN. For each word length k and number of readsN,there are 200 random replicates and AUC values.

To investigate the effect of number of reads, we down sampled the reads from each individual by randomly choosing *N* reads from each sequencing file, where *N* changes from 500 to 4000 by step 500. We then used the same procedure as above to obtain the cross validation AUC. The boxplots of the relationship between AUC values and the number of reads using different word length *k* for SVM and KNN are given in Figs 4 and 5, respectively.

Several conclusions can be drawn from Fig 4 using SVM. First, for all word length *k*, the mean AUC increases with the number of reads when the number of reads is smaller than 3000 and then tends to be stable. For example, when *k* = 6, the mean AUC is 0.90 when the number of reads is 500, while the mean AUC increases to 0.94 when the number of reads is 3000. Second, for a fixed number of reads, mean AUC increases with word length *k*. Similar patterns are observed for KNN (Fig 5) except that the AUC becomes stable at the number of read of 1500. For a given number of reads and word length *k*, the mean AUC based on SVM is higher than that based on KNN.

## Discussion

Several recent studies have clearly shown the advantage of NGS over traditional Sanger sequencing in detecting rare HBV sequence mutations [15] and for the prediction of anti-virus therapy response [31, 32]. In this study, we used high throughput sequencing to investigate composition of HBV sequences in a large number of both CHB and HCC individuals, to compare differences of genetic composition between them, and to predict HCC status using novel word pattern based approaches. Several interesting results were obtained. First, we showed that there was extensive heterogeneity of HBV composition among the individuals based on the NGS data. Almost all the individuals contain some marked fractions of both genotype B and genotype C HBV sequences in Chinese individuals infected with HBV. Previous studies have shown the existence of co-infection of different genotypes of HBV [33–35] and inter-genotype HBV co-infection is the prerequisite of HBV recombination incidence that have been reported broadly [36–38]. Our results highlight the importance of using NGS to study the distribution of different genotypes within individuals.

Second, we used a novel word pattern based approach to cluster the individual samples and investigated the cluster distributions of HCC patients and CHB individuals. Alignment-free sequence comparison based on word counts has been widely used in studying the relationships among sequences or NGS data as reviewed in [39, 40]. However, this approach has not been used for the analysis of HBV data. In this paper, we used alignment-free sequence comparison methods based on word counts to study the relationship among the individuals. We used a dissimilarity matrix based on Manhattan distance between the word frequencies of the NGS data to cluster all the individuals. We showed that there was a strong correlation between the clustering and the fractions of genotypes (B or C) of individuals. This observation was surprising and proved the effectiveness of the alignment-free method on classification based on sequence dissimilarity.

Third, since the second coordinate of PCoA was remarkably correlated with the probability of having HCC, we further applied K-nearest neighbors (KNN) and support vector machine (SVM) approaches to classify HCC or CHB individuals based on word counts. Using cross-validation, we achieved a high area under the receiver operational characteristic curve (AUC) of around 0.88 for KNN and 0.92 for SVM for word length from 4 to 8.

Fourth, we validated the prediction models on an independent set of 46 HCC patients and 31 CHB individuals. The AUC for the independent set was around 0.70 when word length is from 6 to 8 for SVM and 0.67 for KNN. Surprisingly, the AUC for SVM was 0.77 when word length is 3. The good result of *k* = 3 may be explained by the appropriate number of features compared with the number of individuals. The results showed the usefulness of our prediction models for separating HCC patients from CHB individuals. Numerous studies have revealed the divergence in pre-S region between CHB and HCC patients and deletions in pre-S was one of the most noticeable characteristic of HCC patients [41–44]. In addition, fewer studies also found that several nucleotide mutations were also associated with incidence of HCC [19, 45, 46]. Nevertheless, we have succeeded in the establishment of predictive model for HCC via the word pattern frequencies of the pre-S gene following the NGS. The superior performances in both the cross validation and independent cohort validation are also indicative of the advantages of NGS compared with Sanger sequencing.

Finally, we showed that the HCC status can be effectively predicted based on word pattern frequencies using support vector machine and that prediction accuracy increases with the number of reads and becomes stable at about 3000 reads per individual. To our knowledge, this is the first study focusing on the implication of the number of reads on model effectiveness trained on NGS data. With the development of NGS technology, investigators are interested in appropriate number of reads and our study provides guidelines for designing of NGS studies.

Despite these significant results, our study has several limitations. First, the numbers of HCC and CHB individuals, although large compared to previous studies, were still not very large and more individuals are needed to further confirm the applicability of our word pattern based method for investigating HBV infected individuals. Second, the AUC values for the independent test data using both KNN and SVM were much smaller than the corresponding mean AUC values for cross-validation. Potential explanations for the lower AUC value for the independent test data is that the independent samples may come from populations different from that in the training data. Potential experimental variations from the testing data may also decrease the prediction accuracy. Third, we concentrated on the HBV pre-S region in this study and other regions may have different properties. Further studies for other regions or even the whole genome are needed. Fourth, we investigated Chinese HCC and CHB individuals with dominant B and C genotypes. The applicability of our results to other ethnic groups or population samples needs to be further investigated.

In conclusion, our study showed the applicability of word pattern based methods to investigate the diversity of HBV sequences, to compare HBV communities among different individuals, and for the prediction of HCC status. Further studies are needed to extend the results to much larger genomic regions over large number of individuals.

## Materials and methods

### Patient samples and NGS sequencing of the HBV pre-S region

#### Patient samples

We first studied a set of 94 HBV related HCC and 45 CHB patients. We originally planned to recruit about 100 HBV and 100 CHB patients. However, during the process of sampling and sequencing, some samples were discarded due to low concentration of HBV DNA levels (less than 10^4^ IU/ml), failure in amplification, or low number of reads in some files. Most HCC samples were successfully sequenced, while a large fraction of CHB samples encountered sequencing failure. Finally, HCC samples out-numbered CHB samples by a ratio of around 2:1. All the HCC patients received curative hepatectomy (R0) between March 2011 and May 2012 at the Eastern Hepatobiliary Surgery Hospital, Shanghai, China and the diagnoses were confirmed by operative findings and histopathological examination. Tumor tissue samples were collected from HCC patients and serum samples were collected from CHB patients. To validate our predictive models, we additionally enrolled 46 HCC and 31 CHB patients as independent patient cohort and corresponding tumor tissue and serum samples were collected. The Ethics Committee of the Eastern Hepatobiliary Hospital approved this study with approval number EHBHKY2015-01-004 and written informed consent was obtained from all participants.

#### HBV DNA extraction and Illumina sequencing of pre-S region

HBV genomes were extracted from tumor tissue or 200μL of serum samples using QIAamp DNA Mini kit (QIAGEN GmbH, Hilden, Germany) and eluted in 100μL of distilled water. The pre-S region was amplified using Phanta Super-Fidelity DNA Polymerase (Vazyme Biotech, Piscataway, New Jersey, USA) with a pair of primers: 5’-CGCCTCATTYTKYGGGTCA-3’ (forward, nucleotides 2801–2819) and 5’-TCCKGAACTGGAGCCACC-3’ (reverse, nucleotides 62 to 79). PCR amplicons of the pre-S region were purified with Agencourt AMPure XP beads (Beckman Coulter. Beverly, Massachusetts) and quantified with Qubit dsDNA HS assay kit (Invitrogen, Carlsbad, CA, USA). Library of PCR products of the pre-S region was prepared using the TruSeq DNA PCR-Free sample preparation kit (Illumina, San Diego, CA, USA) and run on a MiSeq sequencer (Illumina, San Diego, CA, USA) for paired-end sequencing, according to Illumina’s protocol. Finally, fluorescent signals were analyzed using the MiSeq control software and transformed to paired-end reads with 2*300 bps long sequences. We removed the adapter sequence for each read. To process the raw reads, we first evaluated the quality of raw reads using the online tool fastqc (http://www.bioinformatics.babraham.ac.uk/projects/fastqc/). Then we trimmed the bases at the 3’ end so that all the remaining bases have quality score above 20 (corresponding to error rate 1%) for each read. We also analyzed the sequence data using quality score threshold of 30 (corresponding to error rate 0.1%) resulting in shorter higher standard pair-end reads after quality control, and the results were presented as S1 Supplementary material Tables S9-S11. Next, we joined the paired-end reads with FLASH v1.2.10 (http://ccb.jhu.edu/software/FLASH/) which is widely used in NGS data processing [47, 48]. After that, we removed barcodes from the joint reads and generated sequence data in FASTQ format. The distributions of read length before and after linking were given in S1 Supplementary material FigS6.

### Sequence read genotyping

HBV were divided into ten major genotypes A to J with the dominant genotype B or C in China. Merged pre-S region sequences were genotyped with HBV STAR software [49] that is one of the most widely used software tools for HBV genotyping [50–52]. It is based on a statistically defined, position-specific scoring model (PSSM) [53]. Even though our sequence reads are relatively short compared to the whole genome, it has been shown that any 300 bps sequence segment of the polymerase N-terminal domain containing pre-S is reliable for sequencing-based HBV genotyping [54]. STAR [49] uses all the known HBV sequences with known genotypes to construct a PSSM for each genotype A to H (I and J are not well understood) and then scores each read with respect to each genotype to have eight scores. We further transformed the scores into Z scores as in [49]. As recommended in [49], if the maximum score of a read was above 2.0, we predicted the genotype of the read as the one yielding the highest Z score. If the maximum score was below 2.0, STAR uses a slide window of 150bps to find the genotype for each window. We considered the reads with Z score below 2.0 and having windows with distinct genotypes as recombinant reads.

Consistent with the fact that the dominant HBV genotypes are B and C in China, over 95% of the reads are of the two genotypes or recombinants of B and C for all the samples with some small fractions of genotype A. The fraction of recombinant reads for 95% of the samples (132/139) was less than 5%, and only 3 samples had the fraction of recombinant reads above 20%. Therefore, we ignored the fractions of other genotypes and recombinant reads, normalized the fractions of B and C to sum to 1, and calculated the fraction of genotypes B and C, respectively, for each sample.

In addition to STAR, we also used another program jpHMM [30] for the identification of recombinant reads in NGS reads to see how different programs will affect our results. jpHMM uses a jumping hidden Markov model to identify recombinant reads between different genotypes. For each read, it identifies regions corresponding to a particular genotype. We defined a read to be a non-recombinant if a consecutive region of at least 400bps belongs to the same genotype while only at most 57bps belong to different genotypes. The details were given in the S1 Supplementary material section 2.

### Clustering of individuals based on word pattern frequencies

For each individual, we counted the number of occurrences of any word pattern of length *k* (also called *k*-tuples, *k*-mers, *k*-grams) in the NGS data. The relative frequency of a word of length *k* was its count divided by the total counts of all the words of length *k* for the individual. The distance between any pair of individuals was measured by the Manhattan distance between their corresponding frequency vectors. We constructed a distance matrix of all samples from the training set to see how the individuals cluster together. We chose the Manhattan distance because previous studies showed that it gave better clustering results than Euclidean distance for the clustering of genome sequences in many applications [55]. For different values of *k*, we used principal coordinate analysis (PCoA) to project the data onto two-dimensional space to see how the individuals group together. The basic idea of PCoA was to represent the data in the low dimensional space so that the distances between the samples in the low dimensional space are as close as possible to their true distances. In addition, we hierarchically clustered the individuals based on their word pattern frequencies. We used UPGMA to calculate the distance between any two clusters as the average of all the pairwise distances between the pairs of individuals from both clusters.

### Predicting HCC status using word pattern frequencies

We investigated the optimal approaches for predicting HCC status from the word pattern frequencies. Based on the PCoA and hierarchical clustering results, it can be seen that if the word pattern frequency vector of an individual is similar to others having HCC status, the individual is more likely to have HCC. Therefore, we first used the K-nearest neighbors (KNN) algorithm to predict HCC status, where K is the number of neighbors used for prediction. In KNN, an individual is predicted as having HCC if the fraction of HCC individuals among the top K most similar individuals according to word pattern frequency is above a threshold. We also used supporting vector machine (SVM) to predict HCC status using word pattern frequencies as features. For SVM, we had several kernel functions and parameters to choose from. We used linear kernel only here because for most cases it can work well and it has only one parameter *C*. For the parameter *C*, we used cross validation within the training set to choose *C* yielding the highest AUC (area under the receiver operational characteristic curve) value and used the parameter to construct a model for predicting the testing set.

#### Evaluation criteria and determination of parameters in KNN and SVM

With both KNN and SVM, a score can be obtained based on the word pattern frequency vector of an individual. The higher the score is, the more likely the individual has HCC. Therefore, for a given threshold, we predicted an individual as having HCC if the score was above the threshold and not having HCC if the score was below the threshold. By comparing with the true status of the individuals, we were able to calculate the true positives, false positives, false negatives and true negatives, respectively. The true positive rate (TPR) is the fraction of true positives among the individuals having HCC. The false positive rate (FPR) is the fraction of false positives among the individuals not having HCC. The receiver operational characteristic (ROC) curve shows the relationship between the true positive rate and the false positive rate. The area under the ROC curve (AUC) was used to evaluate the different prediction methods.

We used cross validation within the training set to choose the parameter K for KNN. The cross validation procedures were as follows. From the training set containing 139 samples, we randomly chose 100 samples containing 70 HCC and 30 CHB samples to train a model and predicted the labels of the remaining 39 samples containing 24 HCC and 15 CHB samples. For a given value K, we used the 100 samples to predict the labels of the remaining 39 samples using the KNN method. For each number of neighbors K ranging in 5, 10, 15, ···, 30, we repeated the random separation for 200 times and calculated the mean AUC value for different K. We tried different word length *k* from 2 to 8 and investigated the corresponding results.

The parameter estimation for *C* in SVM was similar to the determination of K for KNN. The separation of the data was the same as that for KNN. For each fixed value of *C* ranging from 2^−5^ to 2^15^, we obtained the SVM classifier using the 100 training samples and obtained the AUC score using the 39 testing samples. We chose the value of *C* yielding the highest average AUC across the 200 separations of the data due to computational time.

Finally, we used the optimal parameters, the number of neighbors in KNN and the value of *C* for SVM, and the complete 139 samples to learn optimal model for predicting HCC. We then evaluated the different approaches using the independent data set.

#### Investigating the effect of the number of reads on prediction accuracy for KNN and SVM

The number of reads can affect the accuracy of predicting HCC. If the number of reads is low, the word pattern frequency vector may deviate from the true composition of the word pattern in the samples resulting in low and highly variant prediction accuracy. In addition, the number of reads can vary for different sources of data. We found that the number of reads of our data varies widely. The difference was quite common due to the experimental technologies and random bias. Therefore, it is important to understand the effect of the number of reads on prediction accuracy. Thus, we conducted the following study to show the relationship between the number of reads and prediction accuracy.

For the data from the training set, we randomly chose N sequences to count the occurrences of word patterns. Here N was chosen to be 500 to 4000 by step 500. If the total number of reads was smaller than N, we just used the entire reads set. We then use the same procedures as above to obtain the AUC scores using both KNN and SVM.

## Supporting information

S1 Table The normalized fraction of genotypes B and C of the individuals used in this study. **The fraction of genotypes is computed using both STAR and jpHMM.** Both training set and independent set of individuals are included.
(XLSX)

S1 Supplementary material Supplementary results including Figs S1-S6 and Tables S1-S11.
(PDF)

S1 Fig The histogram of the fraction of recombinant reads for 139 samples detected by STAR using the threshold Z score of 2 as recommended in Myers et al [49]. (TIF)

S2 Fig **(a) The histogram of the fraction of recombinant reads among the 139 samples using the genotyping tool jpHMM. (b) The relationship between the fractions of genotype B using jpHMM and STAR.** For STAR, we only considered reads having score above 2.0. For jpHMM, only reads with at least 400 bps consecutive region of the same genotype were considered. All fractions were normalized such that the sum of genotypes B and C is 1. Each dot corresponds to a sample.
(TIF)

S3 Fig (a) The histograms of genotype B reads among the 94 HCC patients and 45 CHB individuals genotyped using jpHMM. (b) The relationship between the ratio of the fraction of HCC individuals in the bin over that of the CHB individuals and the fraction of genotype B sequences.
(TIF)

S4 Fig PCoA plots based on the 94 HCC and 45 CHB patients. The distance matrix is calculated based on the Manhattan distance between the frequency vectors of word patterns of length (a) k = 6 and (b) k = 8, respectively. Color shows the fractions of geno-types B and C reads based on the jpHMM genotyping results. Red represents 100% genotype B and blue represents 100% genotype C. Reference B and C sequences are also added on the figures as references. The relationship between the first principal coordinate and the fraction of genotype B calculated using jpHMM, (c): k = 6, (d): k = 8.
(TIF)

S5 Fig The hierarchical clustering results of 94 HCC and 45 CHB samples from the first data set. The samples are colored with four different colors: red means HCC sam-ples with genotype C dominant, yellow means HCC samples with genotype B dominant, green means CHB samples with genotype C dominant, and blue means CHB samples with genotype B dominant. The dominant genotype is defined as the genotype having the largest fraction. The genotype fractions are calculated using jpHMM.
(TIF)

S6 Fig **Histograms of read length:** (a) data trimmed under Q20, (b) data trimmed under Q30. Number of reads in the corresponding files are indicated in the legend.
(TIF)
