# Supplementary material for: Deep sequencing of HBV pre-S region reveals high heterogeneity of HBV genotypes and associations of word pattern frequencies with HCC
Source: PLoS Genet. 2018 Feb 23;14(2):e1007206. doi: 10.1371/journal.pgen.1007206 (PMC5841821; doi:10.1371/journal.pgen.1007206)
Supplement: S1 Supplementary material — Supplementary results including Figs S1-S6 and Tables S1-S11. (PDF) [file pgen.1007206.s002.pdf]

Deep Sequencing of HBV Pre-S Region Reveals High Heterogeneity of HBV Genotypes and Associations of Word Pattern Frequencies with HCC

Supplementary Materials

Xin Bai¶, Jian-an Jia¶, Meng Fang, Shipeng Chen, Xiaotao Liang, Shanfeng Zhu, Shuqin Zhang, Jianfeng Feng, Fengzhu Sun*, Chunfang Gao*

1 Genotype NGS reads using STAR[1]

STAR is a widely used software package that can identify the genotype as well as recombination of a specific HBV sequence read. STAR assigns a read eight normalized Z scores corresponding to genotypes A to H. If the highest Z score is above 2, the read is predicted to have the genotype corresponding to the highest Z score according to the recommendations given in[1]. Otherwise, STAR uses a sliding window approach of 150bps by step 1bp to detect recombination and assigns the read as recombinant from more than one genotypes. For each sample, we calculated the fraction of recombinant reads. Figure S1 shows the histogram of the fraction of recombinant reads for the 139 samples under study. About 95% of the samples (132/139) have the fraction of recombinant reads below 5%, and only 3 out of 139 samples have more than 20% recombinant reads.

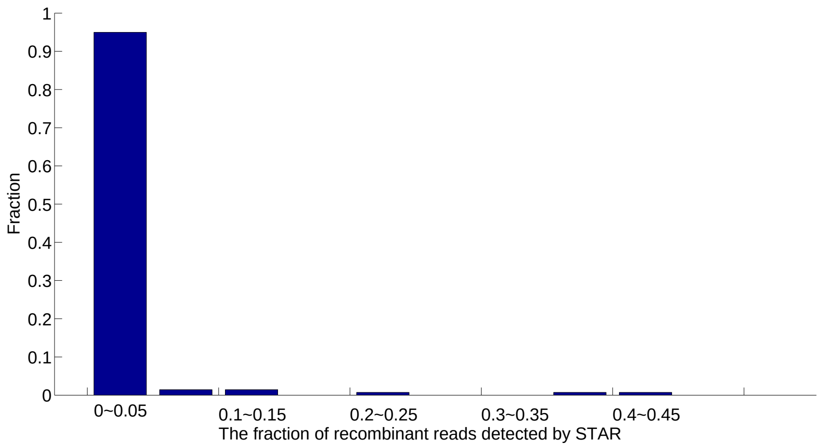

Figure S1. The histogram of the fraction of recombinant reads for 139 samples detected by STAR using the threshold Z score of 2 as recommended in Myers et al. [1] .

2 Genotype NGS reads using jpHMM[2]

In order to see how the results of our study depend on different genotyping software tools, we used another program jpHMM to genotype the NGS reads. jpHMM uses a jumping hidden Markov model to identify regions of a read potentially belonging to different genotypes. The computational speed of jpHMM is much slower than that of STAR. In our study, if the length of a consecutive region of a read having the same genotype is above 400 (the length of the whole region without insertions or deletions in our study is 457 bp), the read is defined as having that genotype. Otherwise, the read is considered as recombinant. For each sample, we calculated the fraction of recombinant reads and plotted the histogram of the fractions of recombinant reads of the 139 samples as shown in Figure 2(a). We also calculated the fraction of reads having genotype B among the reads having genotype B or C and compared with the fraction calculated using STAR as shown in Figure S2(b). It can be seen from the figure that most dots scatter around the line $y = x$ and there are a few dots deviate from $y = x$. Since the results come from different software tools and different criteria were used for genotyping, some differences between the two fractions using STAR and jpHMM were expected. On the other hand, the figure clearly shows that the two fractions from STAR and jpHMM are highly associated and our results are robust and consistent to different genotyping tools.

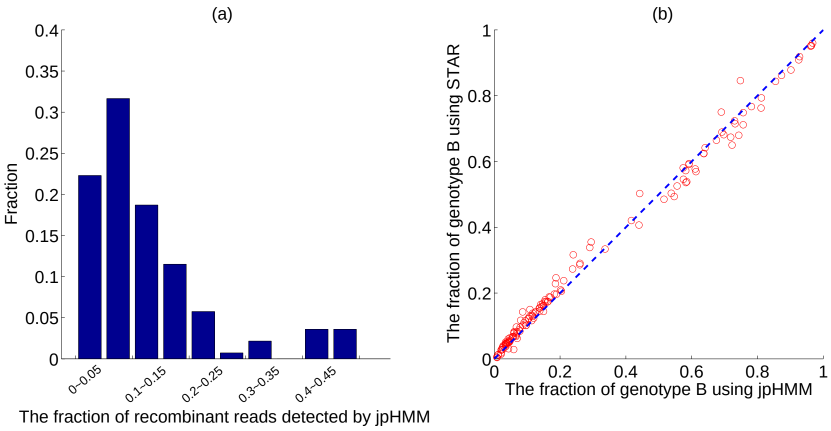

Figure S2. (a) The histogram of the fraction of recombinant reads among the 139 samples using the genotyping tool jpHMM. (b) The relationship between the fractions of genotype B using jpHMM and STAR. For STAR, we only considered reads having score above 2.0. For jpHMM, only reads with at least 400 bps consecutive region of the same genotype were considered. All fractions were normalized such that the sum of genotypes B and C is 1. Each dot corresponds to a sample.

Figure S3(a) shows the histograms of fraction of genotype B reads among the 94 HCC and 45 CHB samples, respectively, and Figure S3(b) shows the relationship between the ratio of the fraction of HCC individuals in the bin over that of the CHB individuals and the fraction of genotype B reads based on the genotyping results using jpHMM. In Figure S3(b), we merged some bins as there are too few samples in those bins, which is the same to Figure 1 in the main text. Again we showed that the probability of having HCC decreases with the fraction of genotype B reads.

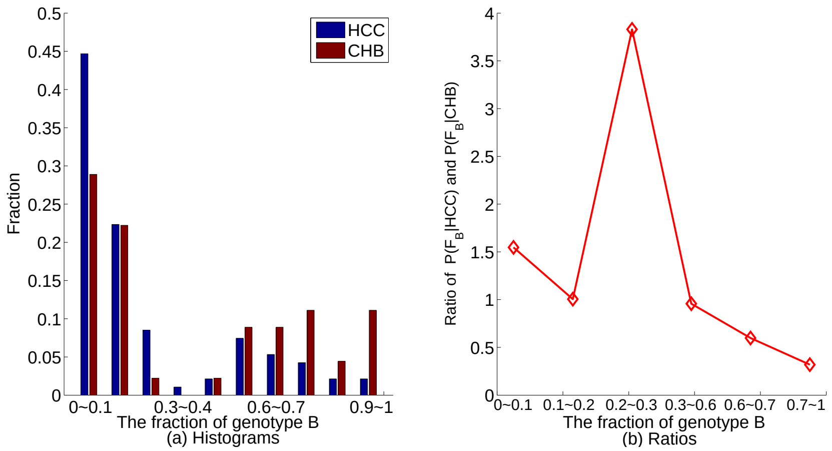

Figure S3. (a) The histograms of genotype B reads among the 94 HCC patients and 45 CHB individuals genotyped using jpHMM. (b) The relationship between the ratio of the fraction of HCC individuals in the bin over that of the CHB individuals and the fraction of genotype B sequences.

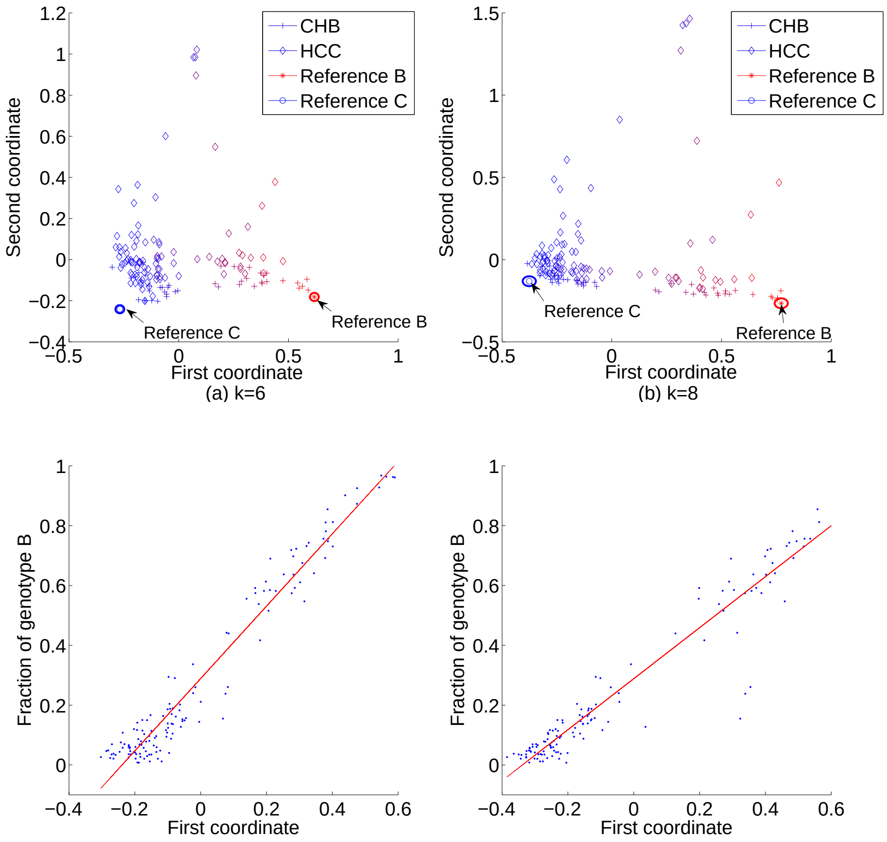

Figure S4. PCoA plots based on the 94 HCC and 45 CHB patients. The distance matrix is calculated based on the Manhattan distance between the frequency vectors of word patterns of length (a) k = 6 and (b) k = 8, respectively. Color shows the fractions of genotypes B and C reads based on the jpHMM genotyping results. Red represents 100% genotype B and blue represents 100% genotype C. Reference B and C sequences are also added on the figures as references. The relationship between the first principal coordinate and the fraction of genotype B calculated using jpHMM, (c): k = 6, (d): k = 8.

Figure S4 (ab) show the PCoA plots of the 139 patients marked with colors indicating the fractions of genotypes B and C calculated using jpHMM. We can see that the first principal coordinate increases with the fraction of genotype B reads. Figure S4 (cd) show the relationship between the first principal coordinate and the fraction of genotype B calculated using jpHMM. It is clear that the dots scatter around the line $y = x$ such that they are highly correlated. The results are consistent with the results shown in Fig 2 in the main text based on STAR.

We further calculated the Spearman and Pearson correlations between the first principal coordinate using PCoA and the fraction of genotype B calculated using jpHMM. The results are shown in Table S2. It shows that both the Spearman and Pearson correlations increase and become stable with the increase of word length k, consistent with the conclusions from Table 1 in the main text based on STAR.

Table S2. The Spearman and Pearson correlations between the first principal coordinate using PCoA and the fraction of genotype B based on jpHMM

Correlation	$k=2$	$k=3$	$k=4$	$k=5$	$k=6$	$k=7$	$k=8$
Spearman	-0.38	-0.19	0.39	0.80	0.89	0.92	0.92
Pearson	-0.39	-0.23	0.48	0.92	0.97	0.97	0.96

Figure S5 shows the clustering results of the 139 samples with colors indicating different dominant genotypes defined based on jpHMM and HCC or CHB status. This figure corresponds to Figure 3 in the main text.

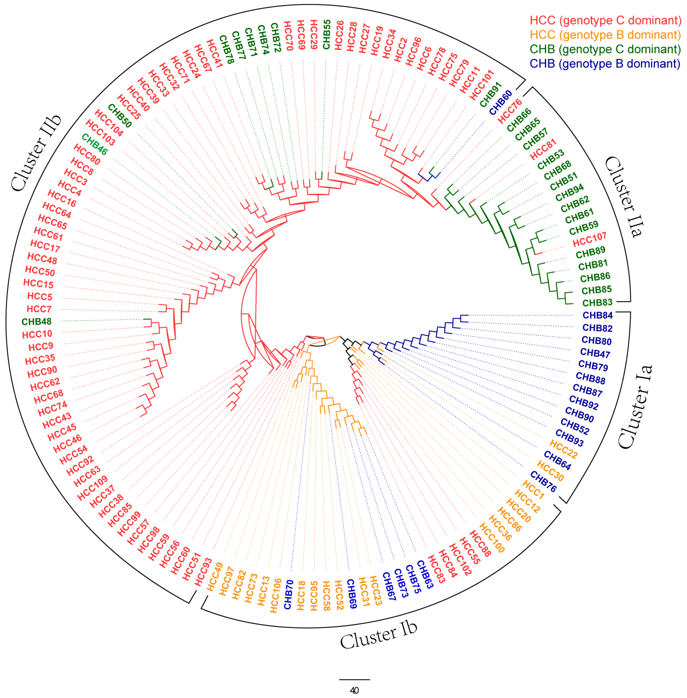

Figure S5. The hierarchical clustering results of 94 HCC and 45 CHB samples from the first data set. The samples are colored with four different colors: red means HCC samples with genotype C dominant, yellow means HCC samples with genotype B dominant, green means CHB samples with genotype C dominant, and blue means CHB samples with genotype B dominant. The dominant genotype is defined as the genotype having the largest fraction. The genotype fractions are calculated using jpHMM.

3 Results based on selecting a subset of words for predicting HCC status

Tables S3-S8 give the prediction results using selected words with different values of $\alpha = 0.05, 0.01, 0.001$, respectively. Although selecting subsets of words can give better results for cross-validation, the prediction results for independent data are worse.

3.1 α=0.05

Table S3. Prediction results from KNN using different word length k and only the selected words based on training set. *CV: cross-validation

Word length k	$k=2$	$k=3$	$k=4$	$k=5$	$k=6$	$k=7$	$k=8$
CV mean AUC	0.86	0.86	0.88	0.89	0.91	0.92	0.92
Predicting AUC	0.62	0.65	0.68	0.67	0.65	0.63	0.61
Optimal K	5	10	10	5	5	5	5
Number of selected kmers	9	19	70	235	803	1707	2365

Table S4. Prediction results from SVM using different word length k and only the selected words based on training set. *CV: cross-validation

Word length k	$k=2$	$k=3$	$k=4$	$k=5$	$k=6$	$k=7$	$k=8$
CV mean AUC	0.86	0.88	0.91	0.93	0.94	0.94	0.94
Predicting AUC	0.64	0.63	0.62	0.69	0.64	0.66	0.64
Optimal C	2^{14}	2^{12}	2^{15}	2^{15}	2^{14}	2^{15}	2^{14}
Number of selected kmers	9	19	70	235	803	1707	2365

3.2 α=0.01

Table S5. Prediction results from KNN using different word length k and only the selected words based on training set. *CV: cross-validation

Word length k	$k=2$	$k=3$	$k=4$	$k=5$	$k=6$	$k=7$	$k=8$
CV mean AUC	0.85	0.86	0.88	0.89	0.91	0.92	0.92
Predicting AUC	0.69	0.65	0.67	0.66	0.62	0.61	0.66
Optimal K	10	15	10	10	5	5	30
Number of selected kmers	8	17	62	207	657	1353	1704

Table S6. Prediction results from SVM using different word length k and only the selected words based on training set. *CV: cross-validation

Word length k	$k=2$	$k=3$	$k=4$	$k=5$	$k=6$	$k=7$	$k=8$
CV mean AUC	0.87	0.89	0.91	0.94	0.96	0.94	0.94
Predicting AUC	0.64	0.66	0.66	0.67	0.63	0.64	0.64
Optimal C	2^{14}	2^{11}	2^{15}	2^{15}	2^{14}	2^{15}	2^{12}
Number of selected kmers	8	17	62	207	657	1353	1704

3.3 α=0.001

Table S7. Prediction results from KNN using different word length k and only the selected words based on training set. *CV: cross-validation

Word length k	$k=2$	$k=3$	$k=4$	$k=5$	$k=6$	$k=7$	$k=8$
CV mean AUC	0.85	0.87	0.88	0.89	0.92	0.92	0.93
Predicting AUC	0.69	0.65	0.73	0.66	0.65	0.65	0.63
Optimal K	10	10	15	5	5	30	10
Number of selected kmers	8	13	46	158	526	946	1038

Table S8. Prediction results from SVM using different word length k and only the selected words based on training set. *CV: cross-validation

Word length k	$k=2$	$k=3$	$k=4$	$k=5$	$k=6$	$k=7$	$k=8$
CV mean AUC	0.87	0.88	0.91	0.94	0.96	0.94	0.95
Predicting AUC	0.64	0.66	0.60	0.66	0.61	0.66	0.65
Optimal C	2^{14}	2^{14}	2^{13}	2^{15}	2^{15}	2^{13}	2^{12}
Number of selected kmers	8	13	46	158	526	946	1038

4 Results based on trimming data using quality score threshold of 30

To see the effect of trimming the reads with different quality score thresholds on our results, we also trimmed the paired-end reads using quality score 30. We then calculated the word frequencies of the samples and re-did the analyses. Table S9 gives the correlation coefficients between the first principal coordinate

of PCoA and the fraction of genotype B reads. Here the Manhattan distances were computed using the new word frequencies of different word length k after trimming the paired-end reads using quality score 30. The fraction of genotype B reads was calculated using STAR. The correlation coefficients increase with word length, consistent with our conclusion shown in Table 1 in the main text based on quality score threshold of 20.

Table S9. The Spearman and Pearson correlation coefficients between the first principal coordinate of PCoA and the fraction of genotype B for the 94 HCC patients and 45 CHB individuals. Different word lengths k were used for computing the Manhattan distance. The fraction of genotype B was calculated using STAR

Correlation	$k = 2$	$k = 3$	$k = 4$	$k = 5$	$k = 6$	$k = 7$	$k = 8$
Spearman	-0.19	0.08	0.13	0.29	0.39	0.51	0.74
Pearson	-0.24	0.12	0.19	0.35	0.49	0.61	0.87

Tables S10-S11 give the prediction results using word frequencies after trimming the paired-end reads using quality score threshold of 30. The prediction accuracies are close to but lower than the results based on quality score threshold of 20 shown in Tables 3-4 in the main text. As quality score 30 is a higher standard that may result in shorter reads, much more information could be lost considering the relatively short region (457 bp) in our study.

Table S10. Prediction results from KNN using different word length k based on quality score 30. *CV: cross-validation

Word length k	$k = 2$	$k = 3$	$k = 4$	$k = 5$	$k = 6$	$k = 7$	$k = 8$
CV mean AUC	0.90	0.88	0.89	0.89	0.89	0.90	0.90
Predicting AUC	0.66	0.67	0.64	0.63	0.66	0.66	0.66
Optimal K	5	5	5	5	5	5	5

Table S11. Prediction results from SVM using different word length k based on quality score 30. *CV: cross-validation.

Word length k	$k = 2$	$k = 3$	$k = 4$	$k = 5$	$k = 6$	$k = 7$	$k = 8$
CV mean AUC	0.89	0.93	0.94	0.95	0.95	0.95	0.94
Predicting AUC	0.56	0.71	0.69	0.62	0.63	0.64	0.64
Optimal C	2^{15}	2^{15}	2^{15}	2^{15}	2^{15}	2^{15}	2^{15}

5 Distributions of read lengths before and after linking

Figure S6 gives the histograms of read lengths before and after linking using quality score threshold of 20 (S6 (a)) and 30 (S6 (b)), respectively. Under the scenario of Q20, the highest bars for R1, R2 and the linked data are 251-300, 151-200, and 451-500, respectively. This shows that the overall quality of R1 reads is higher than that for the R2 reads. About 40% of the paired-end reads were successfully linked. Under the scenario of Q30, most reads in the R1 file still have length 251-300 bps but for the R2 file the most frequent bin is 101-150 bps, which indicates that the reads in the R1 file have relatively higher quality than reads in the R2 file. Under the scenario of Q30, the reads of the R1, R2 and linked files all tend to be shorter as expected, because Q30 is a more stringent criteria for trimming the reads. Also, only about one fourth of the reads are successfully linked.

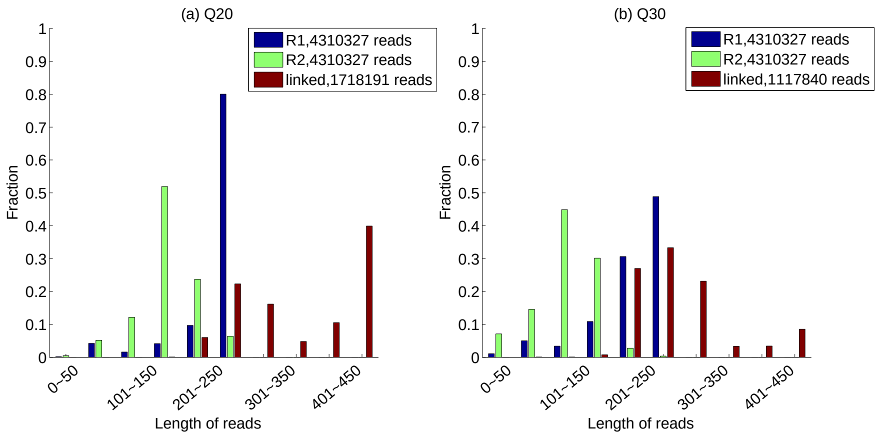

Figure S6. Histograms of read length: (a) data trimmed under Q20, (b) data trimmed under Q30. Number of reads in the corresponding files are indicated in the legend.

References

[1] Myers, R., Clark, C., Khan, A., Kellam, P., and Tedder, R. (2006). Genotyping hepatitis b virus from whole-and sub-genomic fragments using position-specific scoring matrices in hbvstar. Journal of general virology, 87(6):1459–1464.

[2] Schultz, A.-K., Bulla, I., Abdou-Chekaraou, M., Gordien, E., Morgenstern, B., Zoulim, F., Dény, P., and Stanke, M. (2012). jphmm: recombination anal-

ysis in viruses with circular genomes such as the hepatitis b virus. Nucleic acids research, 40(W1):W193–W198.
