# Supplementary figures and images for: Deep sequencing of HBV pre-S region reveals high heterogeneity of HBV genotypes and associations of word pattern frequencies with HCC
Source: PLoS Genet. 2018 Feb 23;14(2):e1007206. doi: 10.1371/journal.pgen.1007206 (PMC5841821; doi:10.1371/journal.pgen.1007206)

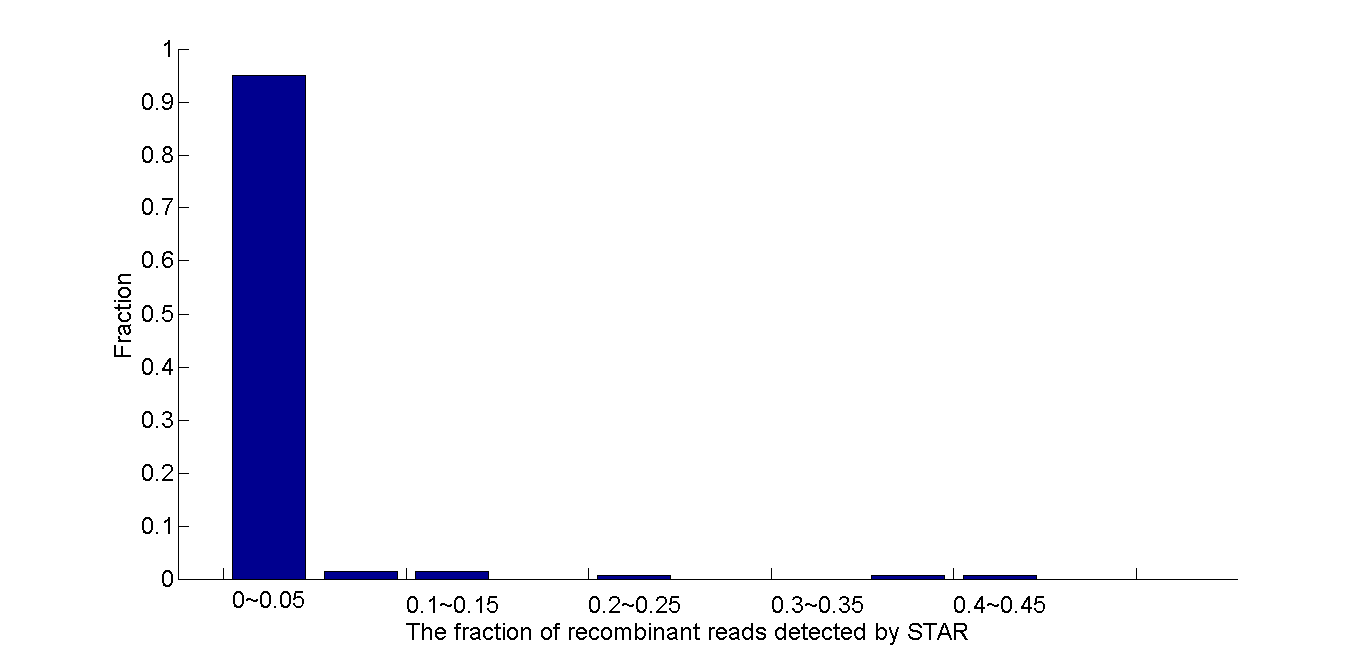

Supplement: S1 Fig — (TIF) [file pgen.1007206.s003.tif]

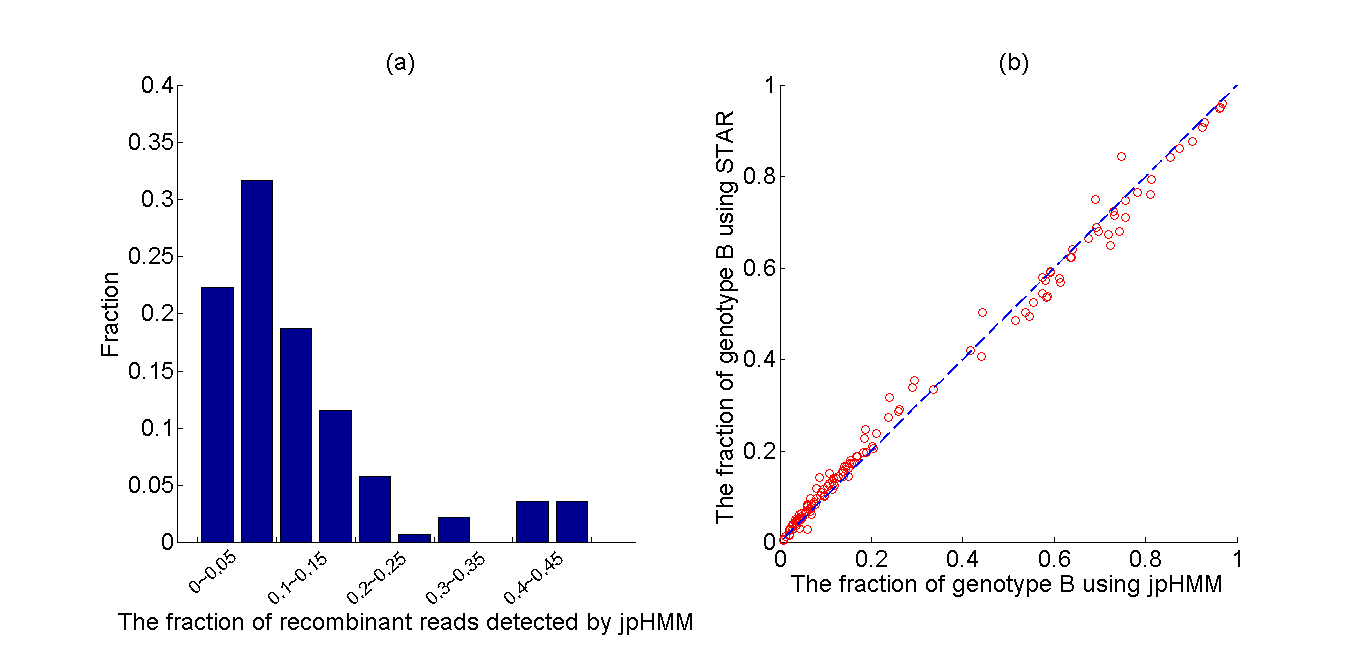

Supplement: S2 Fig — (a) The histogram of the fraction of recombinant reads among the 139 samples using the genotyping tool jpHMM. (b) The relationship between the fractions of genotype B using jpHMM and STAR. For STAR, we only considered reads having score above 2.0. For jpHMM, only reads with at least 400 bps consecutive region of the same genotype were considered. All fractions were normalized such that the sum of genotypes B and C is 1. Each dot corresponds to a sample. (TIF) [file pgen.1007206.s004.tif]

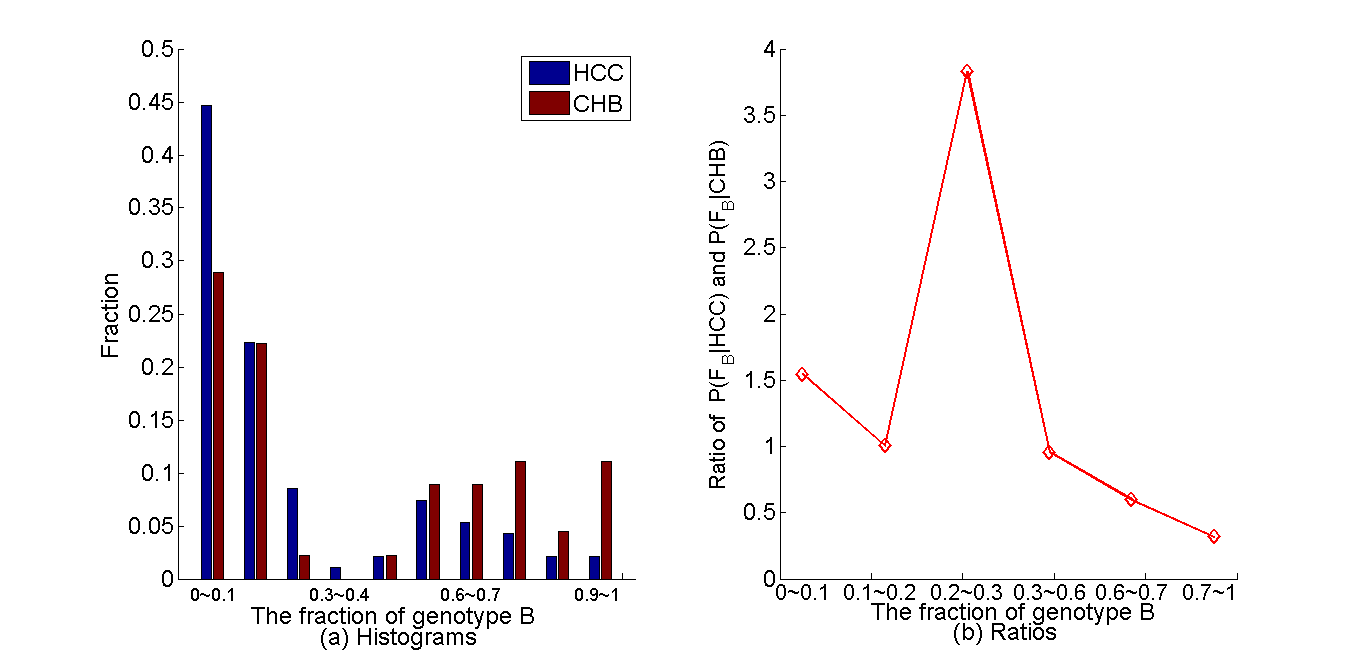

Supplement: S3 Fig — (a) The histograms of genotype B reads among the 94 HCC patients and 45 CHB individuals genotyped using jpHMM. (b) The relationship between the ratio of the fraction of HCC individuals in the bin over that of the CHB individuals and the fraction of genotype B sequences. (TIF) [file pgen.1007206.s005.tif]

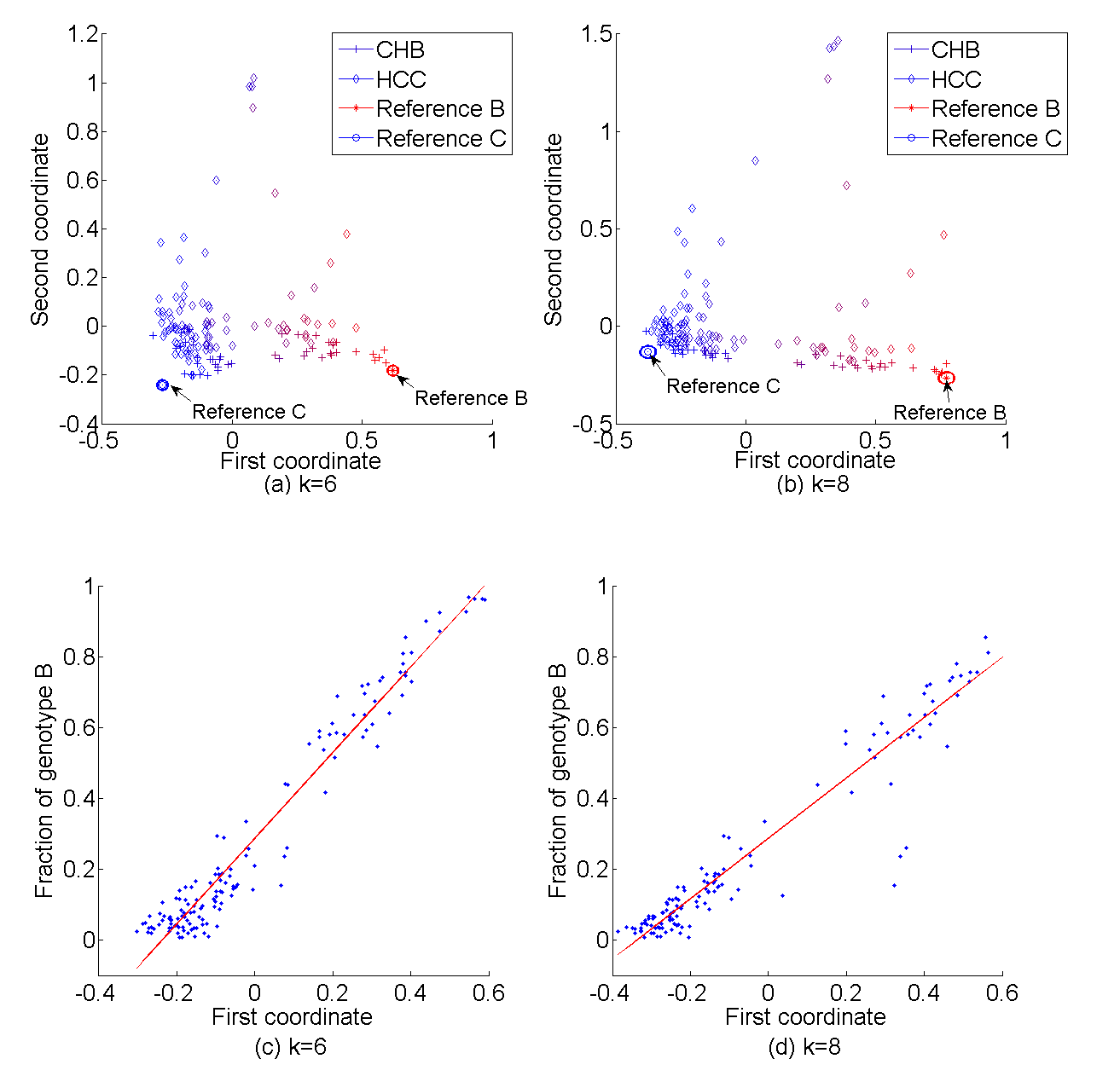

Supplement: S4 Fig — The distance matrix is calculated based on the Manhattan distance between the frequency vectors of word patterns of length (a) k = 6 and (b) k = 8, respectively. Color shows the fractions of geno-types B and C reads based on the jpHMM genotyping results. Red represents 100% genotype B and blue represents 100% genotype C. Reference B and C sequences are also added on the figures as references. The relationship between the first principal coordinate and the fraction of genotype B calculated using jpHMM, (c): k = 6, (d): k = 8. (TIF) [file pgen.1007206.s006.tif]

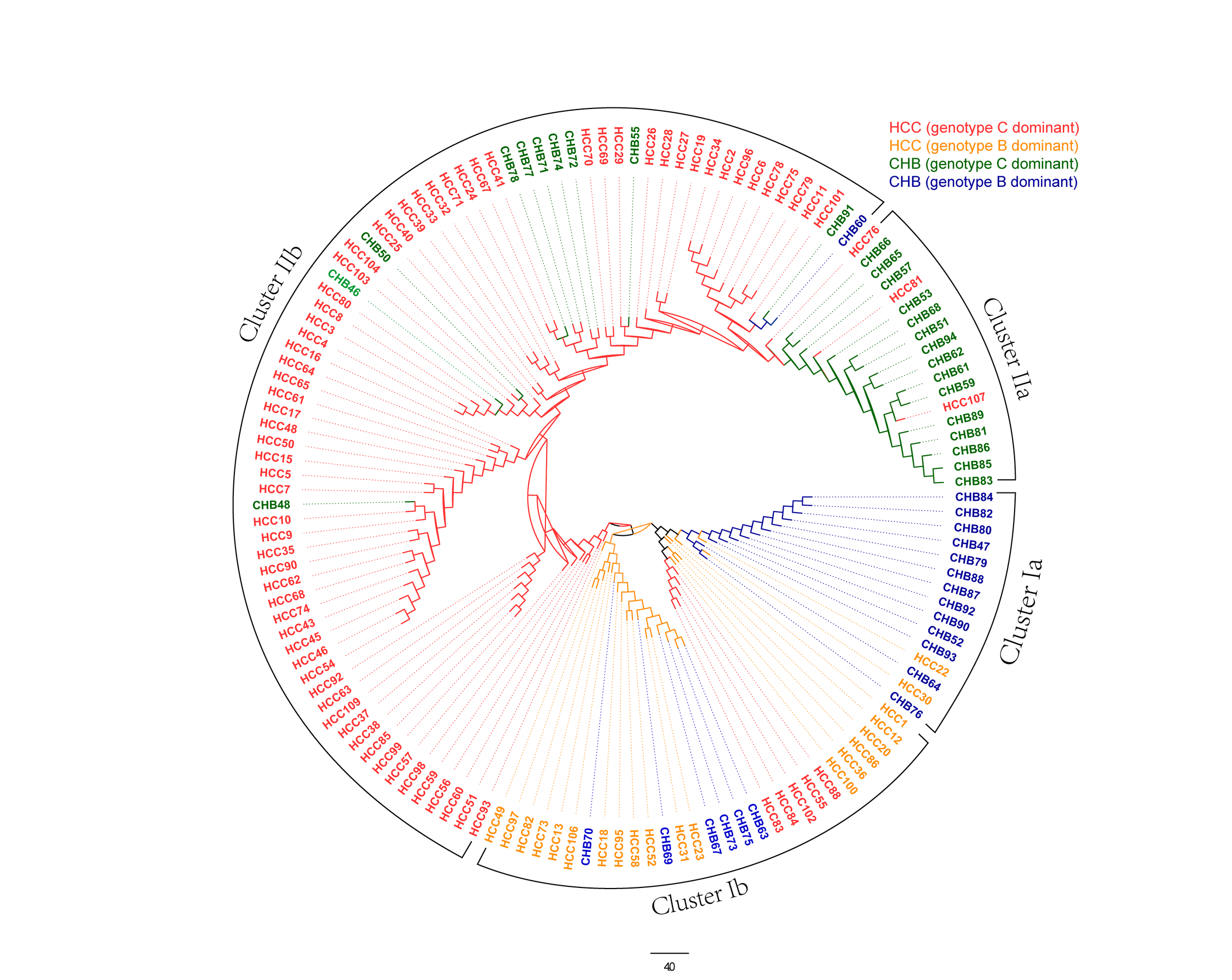

Supplement: S5 Fig — The samples are colored with four different colors: red means HCC sam-ples with genotype C dominant, yellow means HCC samples with genotype B dominant, green means CHB samples with genotype C dominant, and blue means CHB samples with genotype B dominant. The dominant genotype is defined as the genotype having the largest fraction. The genotype fractions are calculated using jpHMM. (TIF) [file pgen.1007206.s007.tif]

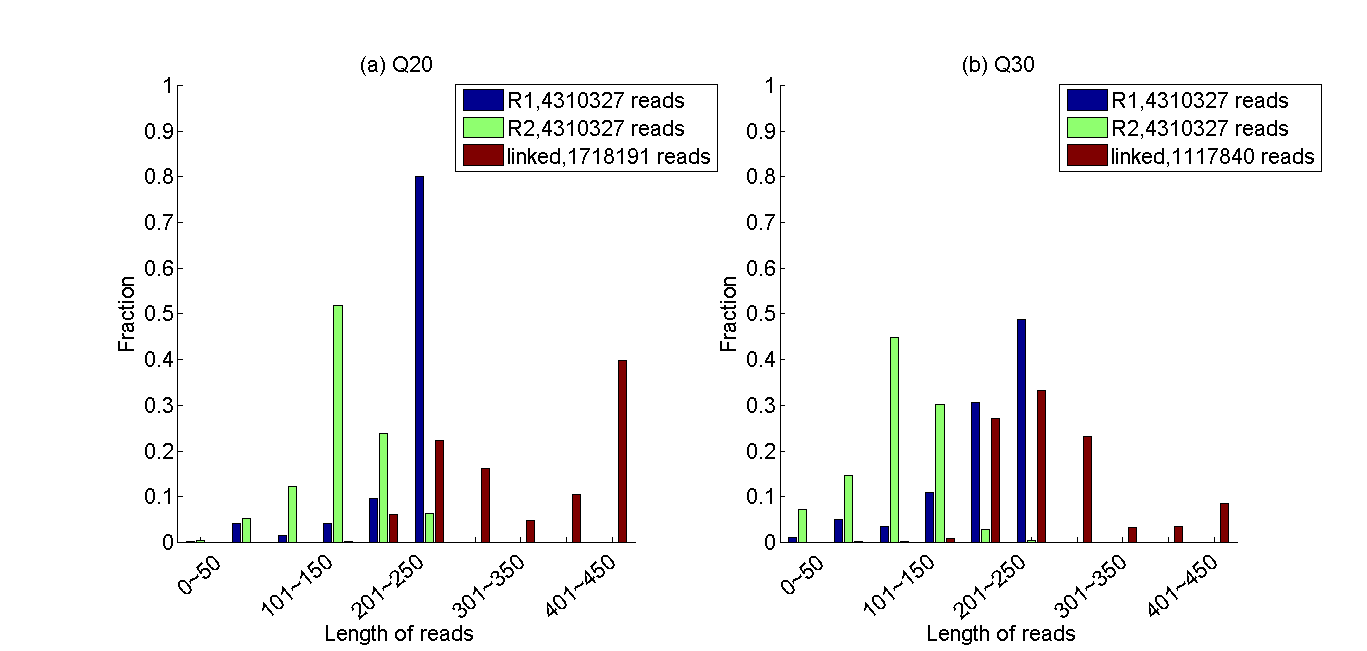

Supplement: S6 Fig — Histograms of read length: (a) data trimmed under Q20, (b) data trimmed under Q30. Number of reads in the corresponding files are indicated in the legend. (TIF) [file pgen.1007206.s008.tif]
